# Biopolymers from Sugar Beet Molasses: Isolation, Characterization, and Bioactive Properties

**DOI:** 10.1021/acsomega.4c09633

**Published:** 2025-03-17

**Authors:** Jenna Crouse, Shad Sellers, Karen Wawrousek, Roberta M. Sabino

**Affiliations:** Department of Chemical and Biomedical Engineering, University of Wyoming, Laramie 82071-0333, United States

## Abstract

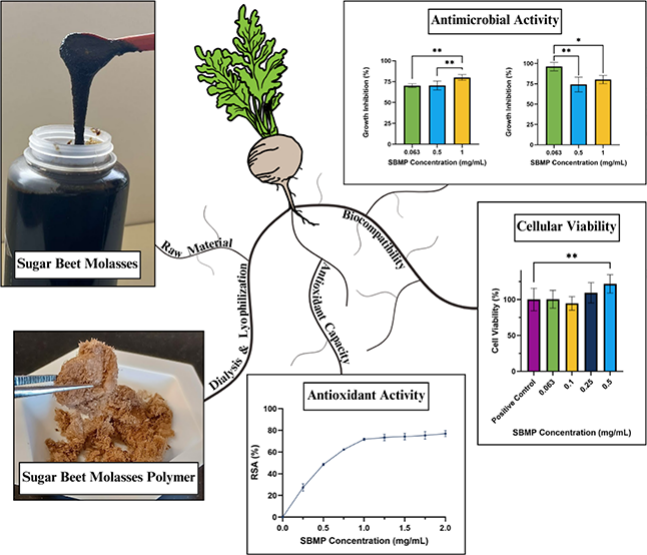

Utilizing the natural biological properties of plant byproducts for a variety of applications presents the opportunity to combine nature’s benefits with sustainable innovation. For this study, sugar beet molasses polymer (SBMP) was isolated from a byproduct of sugar beet processing. The SBMP was analyzed to determine its suitability for potential uses in biomedicine, cosmetics, and antimicrobial coatings. To determine whether the SBMP was indeed a polymer, MALDI-TOF MS was performed. The chemical composition of the SBMP was characterized using XPS, ^1^H NMR, ^13^C NMR, and FTIR. The characterization concluded that the SBMP contains phenolic and hydroxide groups. The presence of these groups was further supported by the SBMP’s high antioxidant activity (∼80% RSA). The SBMP also demonstrated antimicrobial activity against *Rhodococcus erythropolis* (∼80% GI at 1 mg/mg SBMP), *Escherichia coli* (∼80% GI at 1 mg/mg SBMP), and *Saccharomyces cerevisiae* (∼38% GI at 1 mg/mg SBMP). Additionally, the SBMP showed no toxicity to human adipose-derived stem cells (ADSC) at concentrations up to 0.5 mg/mL and supported healthy cellular growth. Due to its strong antimicrobial and antioxidant activity, SBMP could be used in a variety of biomedical, cosmetic, and coating applications.

## Introduction

Biopolymers from natural sources, including chitin and tannins, are materials of interest for environmentally friendly, natural films and coatings^1^ especially since some biopolymers have innate antimicrobial and antioxidant activities.^2^ While biopolymers can be derived from many different sources of plant matter, biopolymers derived from low-value agricultural waste and byproducts are particularly interesting. Plant matter-derived biopolymers can be isolated from inexpensive source materials, their initial isolation may already be completed via other postprocessing procedures, and their isolation does not have to interfere with food production.^3^

Sugar beet processing byproducts are particularly interesting due to their abundance coupled with a lack of valorization strategies. In the United States, approximately 60% of the domestic sugar production comes from sugar beets.^4^ In 2023/2024, it is expected that approximately 5 tons of refined sugar will be produced from a sugar beet crop of approximately 33 million short tons.^5^ The biopolymer isolation and characterization from a liquid byproduct stream called sugar beet molasses (referred to as SBM) is the focus of this work. The SBM is a liquid byproduct stream produced after the final solid and sugar extraction. The SBM is neither a food product nor a precursor to any food product.^6^ It is currently sold at approximately $95/ton, and it is used as an animal feed supplement, fertilizer, and a spray for dust control and deicing of roads.^6,7^

The applications of SBM are currently understudied, presenting a need to fully understand its chemical and biological properties. SBM is a complex mixture known to contain any remaining sugars after processing, as well as organic acids, including amino acids, betaine, raffinose, sucrose, sodium, potassium, chloride, and nitrates.^8^ It is so concentrated that it must be heated to flow through a pipe, and it exhibits high viscosity properties that suggest the presence of biopolymers, as solutions of organic acids, sugars, and ions alone are not expected to be this viscous. It also exhibits a dark reddish-brown color, suggesting the presence of tannins and phenolic compounds, which were also previously detected in sugar beet flesh prior to sugar extraction.^9^ Phenolic compounds from plants can possess bioactive properties such as antioxidant and antimicrobial activity.^10,11^ The SBM color might also hint at the presence of lignins, which are present in extracted sugar beet pulp.^12^ Lignin-derived compounds can produce a reddish hue due to the presence of coniferyl aldehydes^13^ which are natural phenolic phytochemicals that help defend against radical oxidative species.^14^

The diverse variety of potentially extractable biopolymers in SBM, including lignin-derived compounds, tannins, and phenolics, renders it an intriguing material for investigation, especially in the biomaterial sector.^15,16^ One potential application for the biopolymers extracted from SBM is their use as a coating. According to the Centers for Disease Control, indwelling medical devices cause 50–70% of the approximately 2 million healthcare-associated infections.^17^ Some of these infections can be attributed to bacterial growth and the formation of biofilms. Uncontrolled biofouling can lead to undesired cell ingrowth, infections, and eventually device failure.^18^ Since expected compounds in SBM inherently have antimicrobial and antioxidant properties, they may present a promising solution to limiting microbial growth on medical implants. Another potential application for the biopolymers extracted from SBM would be their use in a tissue engineering scaffold. Some plant extracts make excellent scaffolds due to their natural biocompatibility and anti-inflammatory properties.^19^ Inflammation can hinder tissue regeneration, prolonging recovery times.^19^ If the isolated biopolymers in SBM are noncytotoxic, they could serve as a cheap and safe material for constructing new tissue scaffolds. While these examples highlight a few potential applications for the biopolymers extracted from SBM, the possible innate activity of these compounds could provide a cost-effective and abundant raw material for producing biomaterials, coatings, scaffolds, and cosmetics.

The objective of this study was to characterize the biopolymers found in SBM. After the extraction of the SBM biopolymers (referred to as SBMP for sugar beet molasses polymer) via dialysis and lyophilization, the size and chemical composition of the SBMP were evaluated using matrix-assisted laser desorption ionization time of flight mass spectrometry (MALDI-TOF MS), Fourier-transform infrared spectroscopy (FTIR), X-ray photoelectron spectroscopy (XPS), and proton and carbon nuclear magnetic resonance (^1^H and ^13^C NMR). A zeta potential analysis was also conducted to determine the overall charge of the SBMP. Characterization of the biological properties, including antioxidant activity, antimicrobial activity, and cytotoxicity, was conducted to assess potential uses for the SBMP.

## Materials and Methods

### Chemicals

Chemicals were purchased from Sigma-Aldrich unless otherwise noted. The SBM was a kind gift from Wyoming Sugar Company, LLC.

### SBMP Isolation and Preparation

Prior to characterization, the biopolymers were first isolated from sugar beet molasses (SBM) via dialysis and lyophilization. A dilute solution of the SBM (10 mL of SBM in 40 mL of ultrapure water) was dialyzed against ∼4.5 L of ultrapure water at 4 °C using a 7 kDa membrane (Thermo Scientific part #PI68700). The water was exchanged at least 6 times over a 7-day period. The dialyzed sample was collected once the conductivity of the water was below 20 μS/cm. This was done to confirm that most of the small molecules and ions were removed from the bulk mixture. After dialysis, 25 mL aliquots of the dialyzed solution were lyophilized. The fully lyophilized samples were subsequently termed the sugar beet molasses polymer (SBMP). Approximately 0.12 g of SBMP is recovered from 10 mL of undiluted SBM. Lyophilized SBMP was stored at room temperature.

### Characterization of SBMP

#### Matrix-Assisted Laser Desorption Ionization Time of Flight Mass Spectrometry (MALDI-TOF MS)

The MALDI-TOF MS was performed in collaboration with Prof. Franco Basile in the Department of Chemistry at the University of Wyoming to determine the relative size of the SBMP. For data acquisition, the SBMP was diluted to 0.5% w/w in ultrapure water and allowed to stir for ∼2 h. The diluted SBMP was then combined with 1,6-diphenyl-1,3,5-hexatriene (DPH) matrix to produce solutions that contained 1:1, 1:2, and 1:5 ratios of SBMP:DPH.^20^ Then, 1 μL of a 1:1 polyethylene glycol 2,000 (PEG 2,000) and DPH solution was deposited onto a MALDI target plate for calibration purposes. After the calibrant was added, 1 μL of each SBMP:DPH solution was deposited onto the plate and dried under atmospheric conditions for 5 min before analysis with the Sciex TOF/TOF 5800 instrument. Each mass spectrum was collected over two ranges: (1) 7,000–100,000 *m*/*z* and (2) 6,000–170,000 *m*/*z*. MALDI-TOF MS SBMP dilutions were measured on five spots, and each spot was read at least three times. The mass spectra data were analyzed with TOF/TOF Series Explorer Software, mMass open-source software (www.mmass.org, accessed on 27 June 2024), and Origin Software.^21^ For the MALDI-TOF spectra, it was assumed that 1 *m*/*z* was equivalent to 1 Da.^22^

#### Fourier Transform Infrared (FTIR) Spectroscopy

The analysis of the different functional groups present in the SBMP was conducted using Fourier transform infrared spectroscopy (FTIR). The FTIR spectra were obtained with the Alpha II FTIR spectrometer (Bruker, Serial #113127) in the spectral range of 400–4000 cm^–1^, with 19 scans, a resolution of 4 cm^–1^, and in ATP mode. For analysis, a small sample (∼1 mg) of lyophilized SBMP was used. The data were analyzed using OPUS software with a peak threshold of 95%, and the data were graphed in Origin.

#### X-ray Photoelectron Spectroscopy (XPS)

The chemical composition of SBMP was characterized by X-ray photoelectron spectroscopy (XPS, Shimadzu). Prior to analysis, a thin layer of SBMP powder was adhered to carbon tape on an XPS sample bar. The sample bar was then incubated in a vacuum oven for 24 h to remove any volatile components from the SBMP. Survey scan spectra were obtained from 0 to 1200 eV at a pass energy of 80 eV. High-resolution spectra were collected for carbon (C 1s), oxygen (O 1s), nitrogen (N 1s), silicon (Si 2p), and sulfur (S 2p). All of the high-resolution scans were collected with a pass energy of 40 eV. The high-resolution scans of silicon and sulfur were performed to detect the presence of contaminants. The XPS scans were calibrated with the known binding energy of carbon (284.5 eV).^23^ XPS analysis was tested on at least three spots on three different powdered samples. MultiPak and Origin software were used for peak-fit analysis.

#### Nuclear Magnetic Resonance (NMR)

The proton and carbon nuclear magnetic resonance (^1^H and ^13^C NMR) spectra were collected to identify the different chemical bonds present in the SBMP. For the acquisition of both spectra, the SBMP was diluted to 10% w/w in D_2_O (Serial #100341455) and allowed to stir for ∼2 h. The samples were then added to Norell Standard Series 5 mm NMR tubes (lot no. 3110) prior to analysis by the Nuclear Magnetic Resonance Facility at the University of Wyoming. The solution-state NMR spectroscopy data were acquired with a Bruker Avance III 600 NMR spectrometer operating at Larmor frequencies of 600.2 MHz (^1^H) and 150.9 MHz (^13^C). The instrument had a Bruker 5 mm PABBO BB-1H/D Z-GRD probe operating at 25 °C and a Topspin 3.2 (Bruker). The one-dimensional ^1^H NMR spectrum was recorded with a 30° flip angle, a 20 ppm sweep width, and a 1 s relaxation delay. For the ^1^H NMR spectrum, 65536 data points were collected over 128 sweeps. The one-dimensional ^13^C NMR spectrum was recorded with a 30° flip angle, 300 ppm sweep width, 2 s recycle delay, and proton Waltz-16 decoupling. For the ^13^C NMR spectrum, 65536 data points were collected over 27648 sweeps. After the spectra were acquired, the data were analyzed using Origin software.

#### Zeta Potential

Zeta potential, a measurement of the electrical potential at the slipping plane of molecules in a solution, was measured using phase analysis light scattering. A stock solution was prepared by diluting the SBMP in ultrapure water to make a 0.1% w/w solution. The stock solution was then stirred for ∼2 h before further use. After being stirred, the stock solution was diluted with either 0.01 M HCl or 0.01 M NaOH to create seven 0.01% w/w solutions with varying pH levels (3–9). The solutions were placed in 1 cm cuvettes and analyzed in a NanoBrook 173 Plus (Brookhaven Instruments, Serial #270005). Data were collected over 1 s, and the data analysis was completed using the Particle Solution v.3.3 software (Brookhaven Instruments).^24^ The p*K*_a_s of the SBMP were calculated using the pH values corresponding to half the maximum zeta potential (Zeta_max_).^25^ Zeta_max_ was determined by finding the zeta potential plateau, which corresponds to the most-ionized state of the SBMP.^25^ The zeta potential acquisition was repeated three times for each pH value. For all of the solutions, ultrapure water was used for the background measurement.

### Antioxidant Activity

The antioxidant activity of SBMP was analyzed using the free radical 2,2-diphenyl-1-picrylhydrazyl (DPPH) assay, with ethanol replacing methanol.^26,27^ The DPPH stock solution was prepared by dissolving 10 mg of solid DPPH (lot no. U10I018) in 25 mL of ethanol. A stock solution of the SBMP was prepared by dissolving the SBMP in ultrapure water and stirring it for ∼2 h. Then eight serial dilutions (0.25, 0.5, 0.75, 1, 1.25, 1.5, 1.75, and 2 mg/mL) were prepared. In the dark, 100 μL of the DPPH stock solution was added to 100 μL of each SBMP serial dilution in a 96-well plate. In the same well plate, 100 μL of ethanol was added to 100 μL of each SBMP serial dilution. These wells were used as blanks to account for the background color of the SBMP. For the control, 100 μL of the DPPH stock solution was added to 100 μL of ultrapure water. The plate was then incubated in the dark on a shaker plate (235 rpm) for 30 min. After 30 min, the absorbance of the plate was read at 517 nm using a Biotek H1 Synergy microplate reader (Serial #14020714). The DPPH assay was repeated three times with five replicate measurements for each test condition. The background absorbance was repeated three times with three replicate measurements. Ascorbic acid (AC) was used as the reference standard. Five AC solutions (1, 50, 100, 150, and 200 mg/mL) were prepared in ultrapure water and were used to build a standard curve (*R*^2^=0.968). The antioxidant activity of the SBMP was analyzed using AC equivalents (mg/mL) and radical scavenging activity (RSA). The RSA was calculated using eq 1.
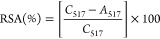
1where *C*_517_ is the average absorbance of the control at 517 nm and *A*_517_ is the absorbance of the diluted SBMP DPPH solution minus the corresponding average background reading of the SBMP dilution at 517 nm.

### Microbial Growth Inhibition

For the growth inhibition (GI) assays, one Gram-positive bacterium (*Rhodococcus erythropolis*), one Gram-negative bacterium (*Escherichia coli*), and one fungus (*Saccharomyces cerevisiae*) were used. Prior to analysis, the bacterial liquid cultures were prepared in M9 medium containing 0.4% glucose,^28^ and the fungal liquid culture was prepared in a synthetic complete dextrose (SCD) medium (1.7 g yeast nitrogen base, 5 g ammonium sulfate, and 20 g dextrose, without amino acid mix).^29^ The *R. erythropolis* and *S. cerevisiae* cultures were incubated at 30 °C, shaking at 150 rpm,^30,31^ and the *E. coli* cultures were incubated at 37 °C, shaking at 150 rpm.^28^ All cultures were incubated for 24 h prior to use in the GI assay. The microbial GI assays were based off the guidelines of the Clinical and Laboratory Standards Institute using the media microdilution method with M9 and SCD media.^27^ The microbial suspensions of *R. erythropolis* and *E. coli* were created by diluting 10 μL of the microbial solutions to an optical density of 0.01. The optical densities of the microbial cultures were measured at 595 nm using a NanoDrop 2000c Spectrometer (Serial #Q685). The microbial suspension of *S. cerevisiae* was created by diluting 10 μL of the microbial solution to ∼1 × 10^6^ cells/mL.^32^ In 96-well plates, 100 μL of diluted microbial solutions were added to 100 μL of the three different SBMP solutions in ultrapure water (0.063, 0.5, and 1 mg/mL). The 96-well plates also included wells that contained 100 μL of diluted microbial solutions and 100 μL of ultrapure water (positive control) and wells with 100 μL of SCD or M9 media and 100 μL of ultrapure water (negative control). Due to the color of the SBMP, wells to account for the background were prepared with 100 μL of the three different SBMP concentrations and 100 μL of SCD or M9 media. Once the plates were prepared, the initial optical density of each well was read at 595 nm using a Biotek Synergy H1 Microplate reader (Serial #2101261F). After the initial reading, the plates were wrapped in parafilm and placed in separate incubators with water dishes at the bottom. The parafilm and water dishes were added to reduce the evaporation of the liquid inside the plates. The plates were incubated for 24 h at 37 °C (*E. coli*)^28^ or 30 °C (*R. erythropolis* or *S. cerevisiae*).^30,31^ After 24 h, the absorbance of each well was measured at 595 nm. The percent GI for each SBMP concentration was calculated using eq 2.
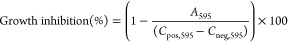
2where *A*_595_ is the absorbance of the culture mixed with SBMP minus the average SBMP background reading for that SBMP concentration at 595 nm, *C*_pos,595_ is the average absorbance of the positive control, and *C*_neg,595_ is the average absorbance of the negative control. All the GI studies were repeated at least three times with five replicate measurements for each test condition and background reading.

### Cell Viability

Human adipose-derived stem cells (ADSC), gifted by Prof. Kimberly Cox-York of the Department of Food Science and Human Nutrition at Colorado State University, were used for both the 3-(4,5-dimethylthiazol-2-yl)-2,5-diphenyltetrazolium bromide (MTT) and alamarBlue assays. ADSC were cultured in 75 cm^2^ surface area tissue-culture polystyrene flasks using MEM growth media (MEM Alpha Modification, ThermoFisher) containing 10% fetal bovine serum and 1% penicillin/streptomycin (henceforth known as cell growth media), and grown in a 37 °C, 5% CO_2_ atmosphere environment.^33^ For the following assays, all of the ADSC used were below passage seven.

The MTT assay was conducted to determine the cytotoxicity of the SBMP, and the alamarBlue assay was conducted to measure cellular proliferation in the presence of SBMP. Prior to running any assay, an SBMP stock solution was prepared by diluting the SBMP in cell growth media to a concentration of 1.2 mg/mL. The stock solution was allowed to stir for ∼2 h. The SBMP stock solution was then sterilized by filtration (0.2 μm, ThermoFisher). The SBMP stock solution was then diluted to the following concentrations: 0.063, 0.1, 0.25, and 0.5 mg/mL SBMP. For both the MTT and alamarBlue assays, 500 μL of each SBMP dilution was combined with 100 μL of a 4.0 × 10^4^ cells/mL ADSC solution in a 24-well plate. The positive control wells contained 500 μL of the cell growth media and 100 μL of the 4.0 × 10^4^ cells/mL solution, and the negative control wells contained 600 μL of the cell growth medium. For the wells that contained the cells, the final cell concentration was 6.7 × 10^3^ cells/mL. The background absorbance of the SBMP was also accounted for by preparing plates that contained the corresponding SBMP concentrations dissolved in ultrapure water. The CyQUANT MTT Cell Viability Assay Kit was purchased from ThermoFisher (Lot #2872018) and performed according to the manufacturer’s protocol with minor modifications. For the MTT assay, the plates were incubated for 48 h before the experiment was conducted. After 48 h, 60 μL of 12 mM MTT stock solution was added to each well in the dark. The plates were protected from light from this point on and incubated for 4 h. Then, 600 μL of the SDS-HCl solution was added to each well and the plates were incubated for another 4 h. The absorbance of each well was then measured at 570 and 650 nm using a Biotek H1 Synergy microplate reader (Agilent Technologies). The cytotoxicity of the different SBMP concentrations was calculated using eq 3.
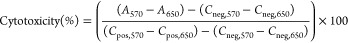
3where *A*_570_ is the absorbance of the diluted SBMP with ADSC at 570 nm minus the corresponding average SBMP background reading at 570 nm, *A*_650_ is the absorbance of the diluted SBMP with ADSC at 650 nm minus the corresponding average SBMP background reading at 650 nm, *C*_neg,570_ is the average absorbance of the negative control at 570 nm, *C*_neg,650_ is the average absorbance of the negative control at 650 nm, *C*_pos,570_ is the average absorbance of the positive control at 570 nm, and *C*_pos_,_650_ is the average absorbance of the positive control at 650 nm.

For the alamarBlue assay, 24-well plates containing ADSC and SBMP were prepared as described above. Plates were incubated for 4 days at 37 °C in a 5% CO_2_ atmosphere, and then, 60 μL of alamarBlue HS Cell Viability Reagent (Lot #2747532, ThermoFisher) was added to each well in the dark. The plates were then incubated for an additional 4 h. The absorbance of each well was measured at 570 and 600 nm using a Biotek H1 Synergy microplate reader (Agilent Technologies). The chemical reduction of alamarBlue was calculated using eq 4.
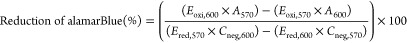
4where *E*_oxi,570_ is 80586, *E*_red,570_ is 155677, *E*_oxi,600_ is 117216, *E*_red,600_ is 14652, *A*_570_ is the absorbance of the diluted SBMP with ADSC at 570 nm minus the corresponding average absorbance of the SBMP background at 570 nm, *A*_600_ is the absorbance of the diluted SBMP with ADSC at 600 nm minus the corresponding average absorbance of the SBMP background at 600 nm, *C*_neg,570_ is the average absorbance of the negative control at 570 nm, and *C*_neg,600_ is the average absorbance of the negative control at 600 nm. After the plates were read, the media was aspirated. The media was then replaced in each well, and plates were placed back in the incubator for four more days. The previously explained alamarBlue procedure was repeated, and the reduction of alamarBlue was recalculated to determine cellular proliferation over time. All of the cell studies were repeated two times with four replicate measurements for each test condition. The background readings were repeated three times with four replicate measurements.

### Statistical Analysis

GraphPad Prism software was used to perform the analysis of variance (ANOVA) and Tukey tests. The statistical differences were compared using (*) for *p* ≤ 0.05, (**) for *p* ≤ 0.01, (***) for *p* ≤ 0.001, and (****) for *p* ≤ 0.0001.

## Results and Discussion

### Characterization of SBMP

#### Matrix-Assisted Laser Desorption Ionization Time of Flight Mass Spectrometry (MALDI-TOF MS)

**Figure 1:**
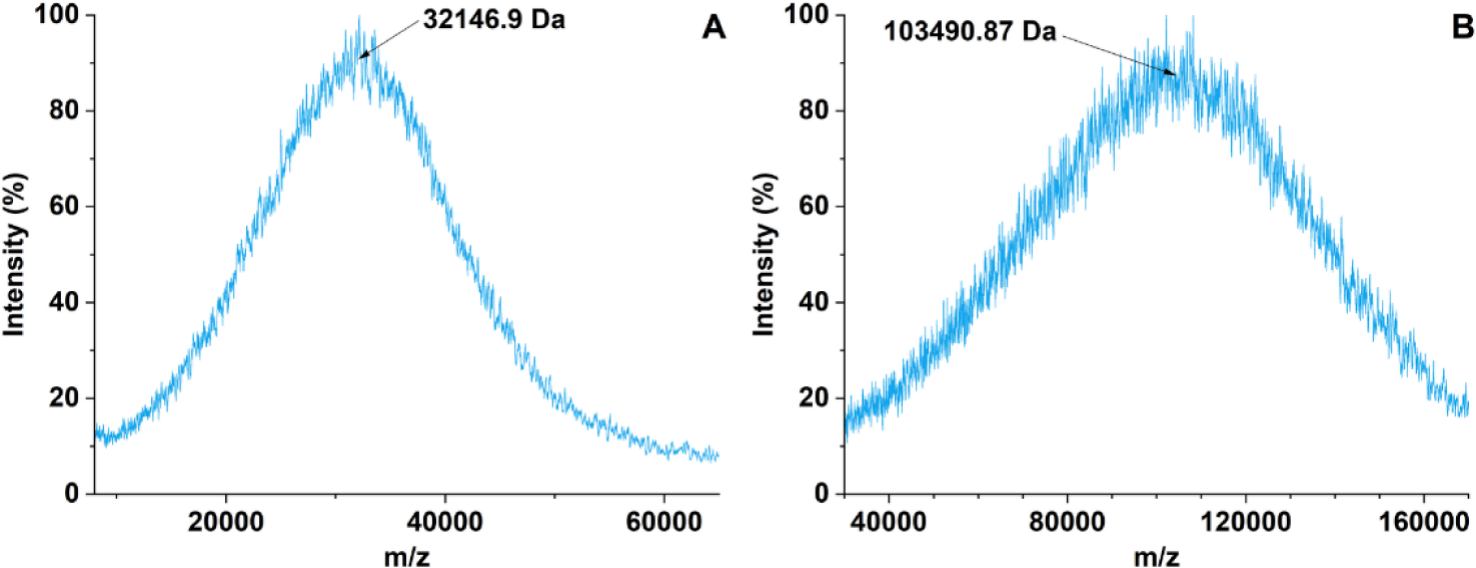
MALDI-TOF mass spectra for SBMP, showing the range from 7,000 to 100,000 *m*/*z* (A) and 6,000 to 170,000 *m*/*z* (B).

One of the key advantages of using MALDI-TOF MS to analyze polymers is that it can measure their average molecular weights. Biopolymers are generally large molecules that can have sizable variations in their molecular weights.^34^ Due to the expected large mass range and possible size variation of potential biopolymers in the SBM, MALDI-TOF spectra of the SBMP were collected over two ranges: 7,000–100,000 *m*/*z* and 6,000–170,000 *m*/*z*. Prior to analyzing the data, the data were denoised with a 15-point Gaussian filter to reduce the effects of the background noise. Figure 1A shows the MALDI-TOF spectrum from the first range with a peak located between 10,000 and 55,000 *m*/*z*, and the apex of the peak located at 32,146.9 *m*/*z*. The peak had a Mw (weighted average molecular weight) of 33,759.1 g/mol, a Mn (number-average molecular weight) of 30,622.5 g/mol, and a polydispersity index (PDI) of 1.1024. For a polymer, the PDI must be less than 1.2 for MALDI results to be considered reasonable. With a PDI of 1.1024, the SBMP has a low enough PDI for the molecular weight estimations to be considered accurate.^35^ A second peak was detected between 35,000 and 165,000 *m*/*z* (Figure 1B) and the apex of this peak was located at 103,490.87 *m*/*z*. The peak had a Mw of 107,517.2 g/mol and a Mn of 97,653.8 g/mol. The PDI for the second peak was 1.1046, which is also below the 1.2 limit. This peak’s broadness is indicative of a wide distribution of different sized macromolecules. The variation of particle sizes is not surprising due to the natural abundance of polydisperse macromolecules.^34^ Besides natural variance, the variation in the SBMP sizes could be attributed to environmental degradation (temperature, humidity, oxygen, light, etc.) or physical reactions with other compounds during the sugar extraction process.^34^ The SBM goes through multiple heating and cooling cycles, and these extreme temperature changes could have produced different-sized molecules.^36^ These reactions/conditions could explain the presence of two different sized molecules in the SBMP sample.

#### Fourier-Transform Infrared (FTIR) Spectroscopy

**Figure 2:**
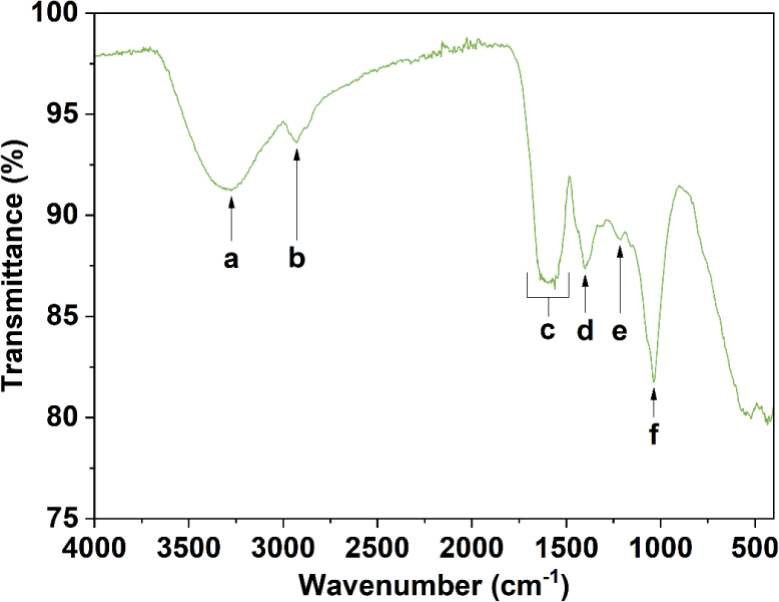
FTIR spectrum of SBMP.

Figure 2 shows the FTIR spectrum for the SBMP. The spectrum contains six main peaks, excluding the fingerprint region, a complex region in which multiple peaks overlap each other. The first region (3000–3750 cm^–1^) contains peak *a* which is located at 3275.6 cm^–1^. This broad peak represents hydroxyl groups including the ones present in phenolic compounds.^37^ The second region (2500–3000 cm^–1^) contains peak *b,* which is located at 2929.0 cm^–1^. This peak represents carbon–hydrogen and oxygen–hydrogen single-bond stretching present in alkanes and carboxylic acids.^37^ The third region (1500–1750 cm^–1^) contains three peaks (1560.7 cm^–1^, 1596.6 cm^–1^, and 1637.7 cm^–1^) all labeled *c* on the spectrum. The peaks at 1560.7 cm^–1^ and 1596.6 cm^–1^ represent aromatic carbon–carbon double-bond stretching and amine nitrogen–hydrogen single bond bending.^37^ These peaks could also represent asymmetric and symmetric COO- stretching.^38^ The peak at 1637.7 cm^–1^ represents carbon–oxygen double-bond stretching.^38^ The fourth region (1100–1500 cm^–1^) contains peak *d* at 1399.9 cm^–1^ and peak *e* at 1214.1 cm^–1^. These peaks represent carbon–hydrogen single bond stretching.^38^ The last region (750–1100 cm^–1^) contains peak *f* at 1036.7 cm^–1^, which represents a carbon–oxygen single bond in an ester.^39^ The FTIR spectrum for the SBMP contains similarities to the FTIR spectra of polysaccharides, lignins, and tannins. All three compounds share the broad hydroxyl peak with the SBMP.^40−42^ For lignins and tannins, this peak also represents the hydroxyl groups present in their phenolic compounds.^40,41^ These groups also share peak *b* with the SBMP, which represents the carbon–hydrogen single bond stretching in the respective compounds.^40−42^ Both tannins and lignins also share the multiple peak region (1400–1600 cm^–1^) with the SBMP, which represents the carbons in aromatic rings.^40,41^ All three compounds also share peaks within the 1000–1300 cm^–1^ range, which represent the carbon–oxygen single bond stretching. The FTIR spectrum of the SBMP provides evidence that its chemical composition closely resembles lignins, tannins, and polysaccharides.

#### X-ray Photoelectron Spectroscopy (XPS)

**Figure 3:**
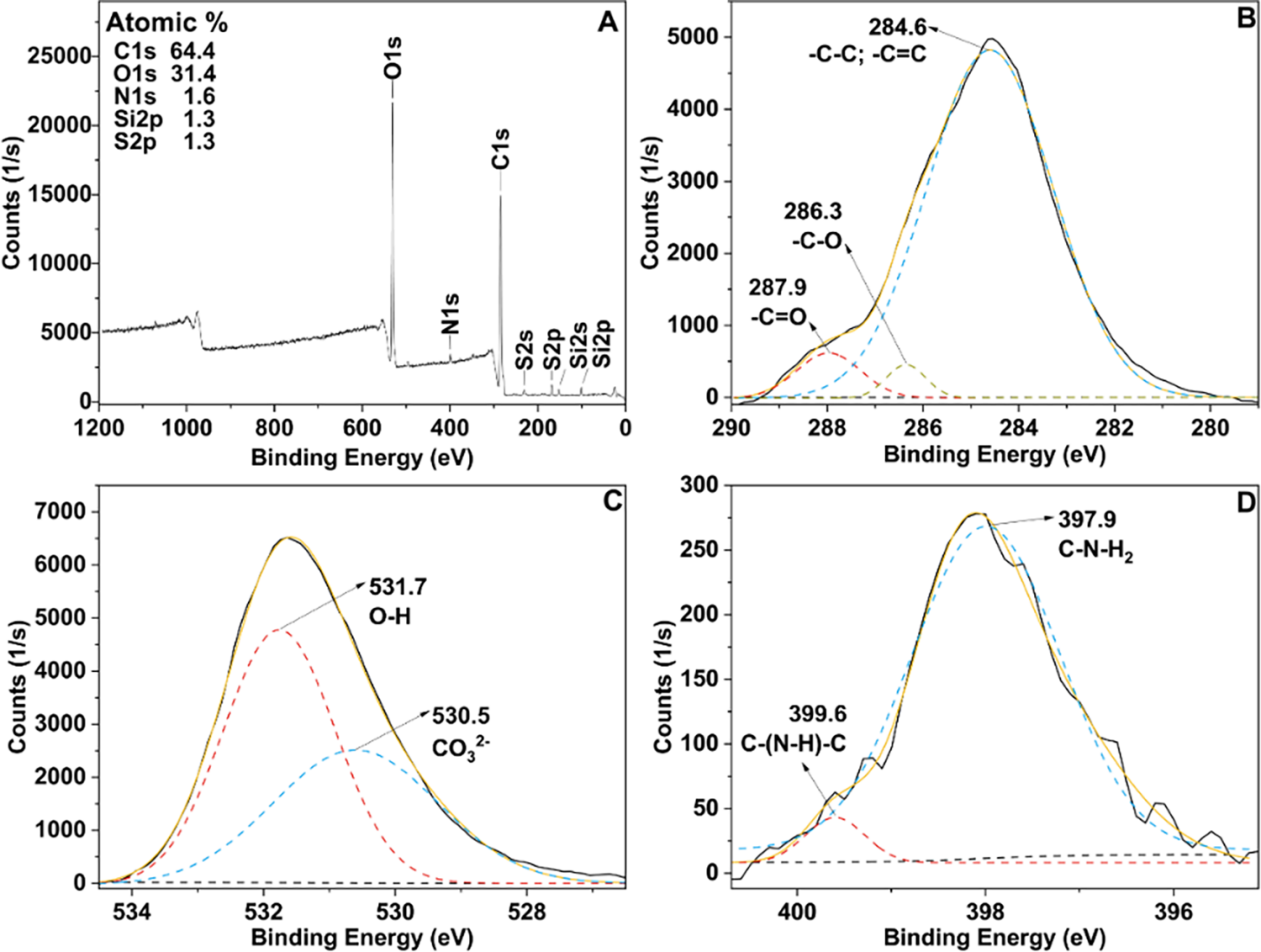
XPS survey scan (A) and high-resolution spectra for C 1s (B), O 1s (C), and N 1s (D) of SBMP.

XPS scans were collected to analyze the elemental composition and identify the different covalent bonds present in SBMP. To accomplish this, both a survey scan (Figure 3A) and three high-resolution scans (Figure 3B–D) were performed. The survey scan contains seven peaks, indicating that SBMP is primarily composed of O (O 1s at 531.3 eV), N (N 1s at 399.0 eV), and C (C 1s at 284.7 eV). Of these, carbon comprised the largest percent area of 64.4% in the scan. Small amounts of S (S 2s at 231.9 eV and S 2p at 168.0 eV) and Si (Si 2s at 152.1 eV and Si 2p at 101.4 eV) were detected. Silicon and sulfur only comprised 1.3% of the total percent area of the peaks, indicating either a very small amount of these elements or that these elements are contaminants from small particles of soil not fully removed in the sugar extraction process. High-resolution scans of both Si and S confirmed that these elements were present at levels consistent with contaminants, and they were excluded from future analysis (data not shown).

The carbon high-resolution scan showed the presence of carbon–carbon single bonds and carbon–carbon double bonds, carbon–oxygen single bonds, and carbon–oxygen double bonds (Figure 3B).^43^ The oxygen high-resolution scan further supports these conclusions by showing the presence of carboxyl groups, represented by the CO_3_^2–^ in the scan (Figure 3C).^44^ Lastly, the high-resolution nitrogen scan showed the presence of primary^23,45,46^ and secondary amines^23,45^ (Figure 3D). The results from the XPS high-resolution scans confirm the findings from FTIR analysis (Figure 2). The presence of alkanes, alkenes, carbon–oxygen double bonds, carbon–oxygen single bonds, and carbon–nitrogen single bonds was also detected in the FTIR spectrum. The detected chemical bonds from the XPS high-resolution scans and the FTIR spectrum suggest the presence of carboxylic acids, amine groups, hydrocarbon chains, and hydroxide groups.

#### Nuclear Magnetic Resonance (NMR)

**Figure 4:**
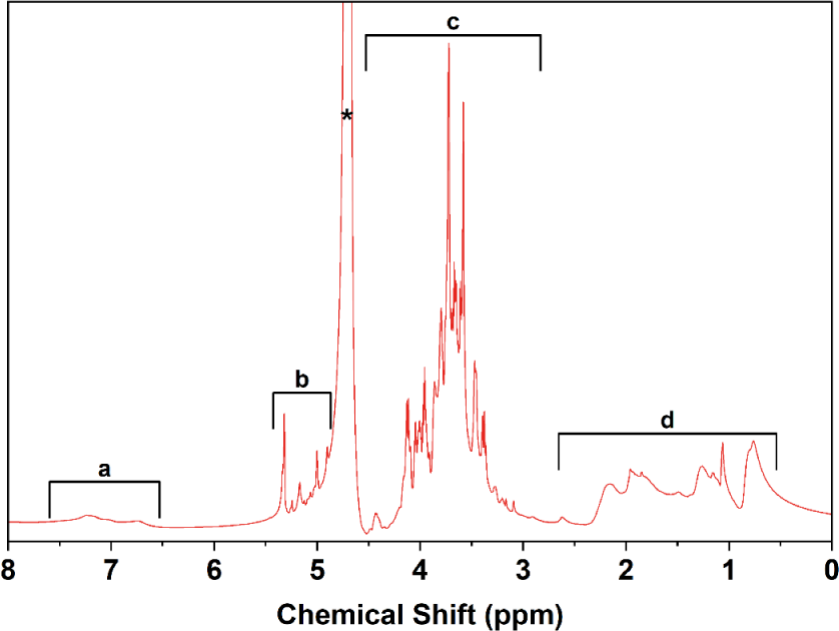
^1^H NMR spectrum of SBMP.

The ^1^H NMR spectrum of SBMP had four distinct peak regions (Figure 4). Region a (6.7–7.6 ppm) contains two small broad peaks that are consistent with the chemical shift of protons in aromatic rings.^47^ The peaks in region b (4.9–5.6 ppm) represent protons connected to double-bonded carbons and aldehyde protons.^47^ The peaks in region c (2.7–4.4 ppm) correspond to a multitude of protons in different chemical bond environments, including amine protons, heterocyclic protons, protons bonded to carbon with an oxygen single bond, and protons adjacent to double-bonded carbons.^47^ The peaks in region d (0.1–2.7 ppm) are within the chemical shift range of saturated hydrocarbon protons.^47^ The peak denoted with an asterisk (4.8 ppm) was produced by the solvent D_2_O.^48^ The ^1^H NMR spectrum confirmed the presence of various bonds identified in the high-resolution XPS scan (Figure 3B–D) and FTIR spectrum (Figure 2). The hydroxide group present in the XPS oxygen high-resolution scan (Figure 3C) was not confirmed with the ^1^H NMR spectrum since D_2_O was used as the solvent.^49^ The detected proton environments were not those expected for pure tannins, polyphenolics, and lignin breakdown products, suggesting that these compounds, if present, may have undergone chemical modifications during the sugar extraction processing. Alternatively, they could be part of a complex mixture with other types of macromolecules, such as polysaccharides and proteins.

**Figure 5:**
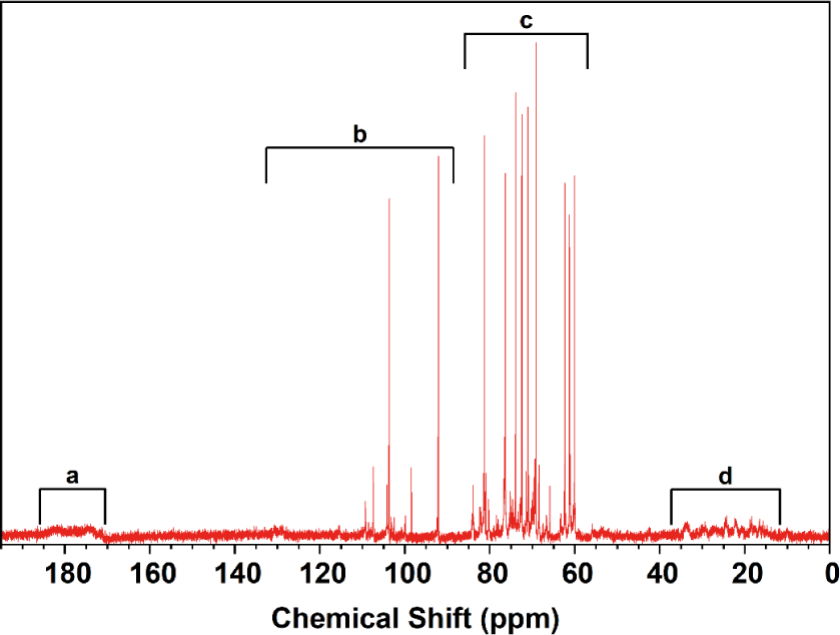
^13^C NMR spectrum of SBMP.

The ^13^C NMR spectrum of SBMP was also separated into four distinct peak regions (Figure 5). Region a (174.0–182.1 ppm) contains small broad peaks that represent carboxyl groups, phenols, or acyl oxygen bound to a ring.^50,51^ The peaks in region b (92.1–130.7 ppm) represent double-bonded carbons and carbons in aromatic rings,^50^ while those in region c (60.1–83.9 ppm) correspond with carbon–oxygen single bonds and carbon–nitrogen single bonds.^50^ Peaks in region d (16.5–33.7 ppm) represent carbons in saturated hydrocarbon chains.^50^ The high-resolution XPS scans (Figure 3B–D) confirmed all the bonds present in the^13^C NMR spectrum and the FTIR spectrum (Figure 2) confirmed the majority of the carbon functional groups. Higher chemical shifts are associated with electron-withdrawing environments, while lower chemical shifts are associated with electron-donating environments. Based on this, the presence of specific chemical groups such as carboxyl (withdrawing) and amines (donating) is suggested.^52,53^

Both the ^1^H NMR and ^13^C NMR spectra confirm the presence of aromatic rings, amines, carbon–carbon double bonds, carbon–oxygen single bonds, and saturated/unsaturated hydrocarbon chains. The ^1^H NMR spectrum also indicates the presence of polysaccharides due to the abundance of narrow peaks within the 3.0–5.5 ppm range.^54^ The conglomeration of polysaccharides in the solution would contribute to the SBMP’s high molecular weight. Additionally, the functional groups identified from both spectra also suggest the presence of lignins. Like lignins, the SBMP contains aromatic rings, carbon–oxygen single bonds, carbon–carbon double bonds, and methoxylated groups.^55^ Lignins also have phenylpropanoids, a group of molecules with aromatic carbon rings and oxygen groups, which could be present in the SBMP. The presence of these different types of bonds and functional groups is consistent with other phenolic, lignin, and tannin-like plant materials.^55−57^

#### Zeta Potential

**Figure 6:**
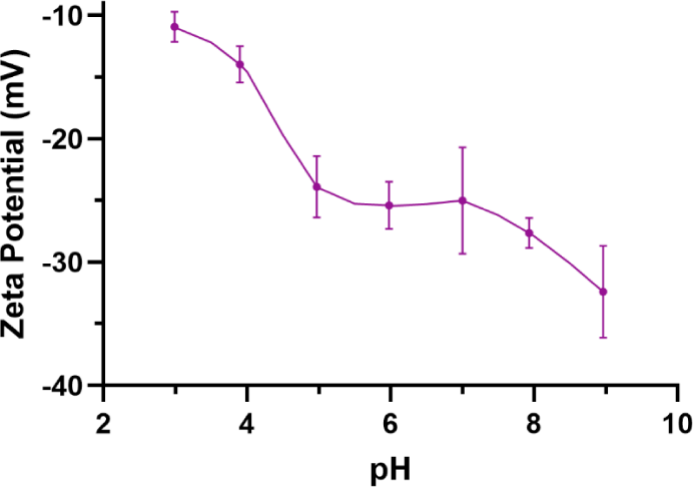
Zeta potential of SBMP at varying pH values. Values represent the mean ± standard deviation (*n* = 3).

Zeta potential was measured as an indication of the surface charge of the SBMP. The zeta potential of the SBMP varied from −10.9 mV at pH 3 to −32.4 mV at pH 9 (Figure 6). Between a pH of 5.5 and 7, the SBMP had a stable zeta potential of approximately −25.3 mV. The overall trend in Figure 6 is consistent with other weakly acidic polymers.^25^ At lower pH, the ratio of [HA] to [A^–^] is high because the acidic environment favors protonation. As the pH increases, the ratio of [A^–^] to [HA] increases due to the deprotonation of the acid.^25^ The data in Figure 6 were also used to calculate Zet*a*_max_ which was used to find the p*K*_a_ values of the SBMP. Two distinct p*K*_a_ values were found: ∼3.6 and ∼10.1.^25^ The first p*K*_a_ value corresponds to the p*K*_a_ of carboxyls (p*K*_a_ 3–4)^58^ and the second p*K*_a_ value corresponds to the p*K*_a_ of phenols (∼10).^59^ These functional groups were confirmed by FTIR, XPS, and NMR.

The charge of the SBMP will be crucial in determining its suitability for various biomedical, antimicrobial, and food-safe coatings.^60,61^ For example, in layer-by-layer (LbL) deposition of polyelectrolyte multilayer (PEM) coatings, alternating layers of polycations and polyanions are deposited onto a charged surface. The electrostatic interactions between these layers help to form a stable coating. PEM coatings can be designed to promote or prevent cell adhesion and inhibit bacterial growth by adding a bioactive compound within the layers.^62^ Therefore, any inherent cell proliferation or antibacterial activity of the SBMP could make it a suitable candidate for use in LbL PEM coatings. Given its overall negative charge, likely due to the presence of carboxyl and hydroxide groups,^63^ the SBMP could serve as the polyanion within the PEM coating.

### Antioxidant Activity

**Figure 7:**
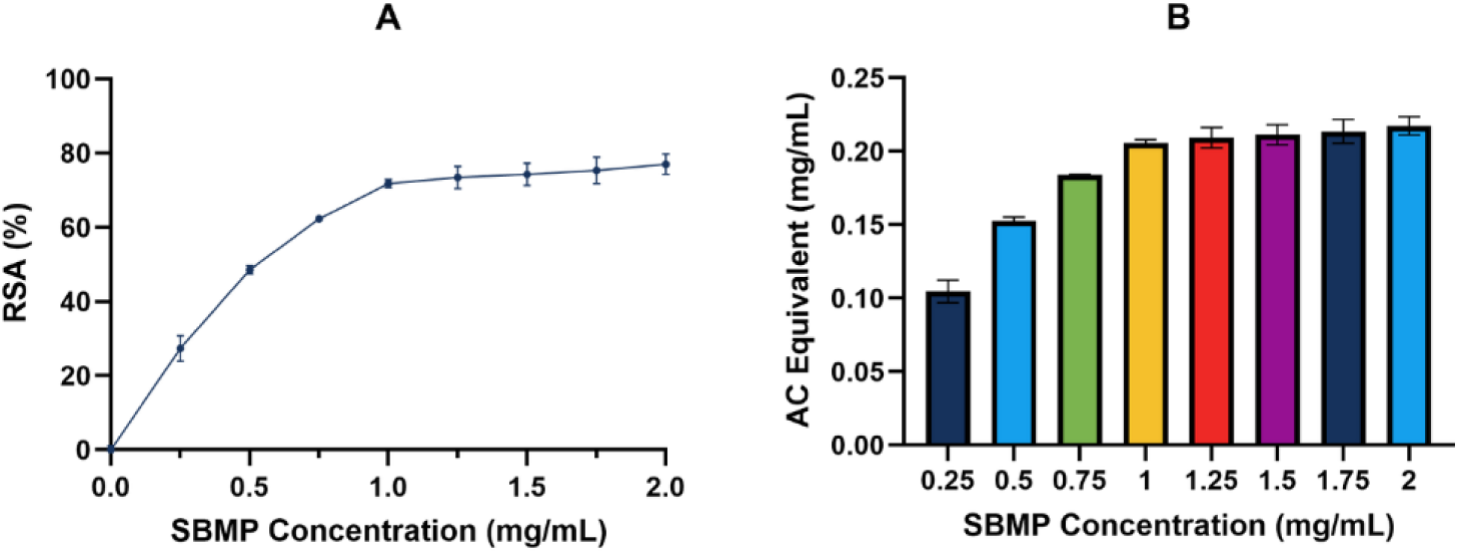
Antioxidant activity of SBMP at different concentrations (0.25, 0.5, 0.75, 1, 1.25, 1.5, 1.75, and 2 mg/mL): radical scavenging activity (RSA) of DPPH (A) and ascorbic acid (AC) equivalence (B). Values represent the mean ± standard deviation (*n* = 5).

Antioxidants are known for their ability to protect cells against free radicals, which play a critical role in the development of different conditions, such as heart disease, inflammation, and cardiovascular diseases. Natural antioxidants are generally plant-derived, and an extract of sugar beet peels has been found to have high antioxidant activity.^64^ The antioxidant activity of the SBMP was assessed by measuring the oxidation of the chemical DPPH in the presence and absence of the SBMP. The measured radical scavenging activity (RSA) was then compared with that of ascorbic acid. The radical scavenging activity of the SBMP increased with concentration until ∼80% RSA was achieved in solution (Figure 7A). The ascorbic acid equivalence for each SBMP concentration was calculated using the standard curve (*R*^2^=0.968).^27^ The SBMP at 1 mg/mL or higher had an RSA equivalent of 0.22 mg/mL of ascorbic acid (Figure 7B). The RSA of the SBMP is relatively high compared to other plant extracts. Other reported plant extracts’ RSAs are much lower, such as diluted *Ficus religiosa* leaf extract, which has an RSA of ∼43%.^65^*Dalbergia sissoo* leaf extract, which is known to contain tannins and coumarins, has a slightly higher RSA (86.3%) than the SBMP. *Dalbergia sissoo’s* high RSA is attributed to the presence of tannins, and it is plausible that possible tannins in the SBMP could contribute to its high RSA.^66^ Lignins, which may also be present in SBMP, exhibit high antioxidant activity due to their phenolic groups. The methoxyl groups and conjugated double bonds in lignins help stabilize phenoxyl radicals, which improves their overall antioxidant potential.^67^ Extracted organosolv lignins from eucalyptus, for instance, have almost an equivalent RSA to the SBMP of ∼79%.^68^ Similar to these extracts with high RSAs, SBMP is expected to contain lignins and tannins, which contain phenolic rings. Additionally, the high RSA could also be attributed to the presence of carboxyl and amine groups,^69^ as both functional groups are good radical scavengers that would enhance the antioxidant activity of the SBMP.

### Microbial Growth Inhibition

**Figure 8:**
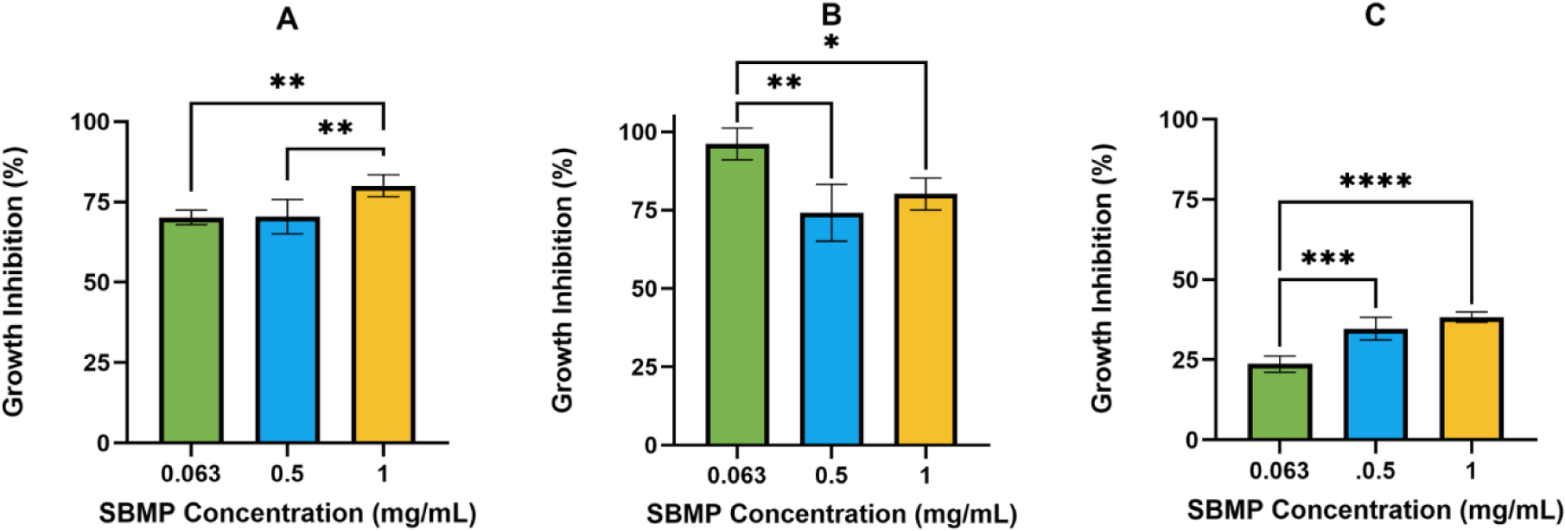
Antibacterial and antifungal activity of the SBMP at different concentrations (0.63, 0.5, and 1 mg/mL) against: *Rhodococcus erythropolis* (A), *Escherichia coli* (B), and *Saccharomyces cerevisiae* (C). Values represent mean ± standard deviation (*n* = 5). *p ≤ 0.05, **p ≤ 0.01, ***p ≤ 0.001, and ****p ≤ 0.0001.

The Gram-negative bacterium *E. coli* is commonly found in the human gastrointestinal tract; however, outside the gastrointestinal tract, it can result in extraintestinal illnesses. *E. coli* is also known for its ability to form biofilms on medical devices, which can result in implant failure. Medical implant-associated infections have also occurred with fungi and Gram-positive bacteria.^71^ Pathogens in biofilms have higher resistance to a host’s immune response and higher tolerance to antibiotics.^17^ Due to these potential resistances, it is important for implanted medical device surfaces to effectively prevent microbial proliferation. For this study, the antimicrobial properties of the SBMP were analyzed with *E. coli* and *R. erythropolis*. These bacteria were selected because they are known to form biofilms.^70,72^ Additionally, *S. cervisiae* was chosen to measure the antifungal activity of the SBMP because it is a model organism, and it could provide initial insight into the SBMP’s antifungal activity.^73^ The GI of each bacterium/fungus was measured in the presence of three SBMP concentrations (0.063, 0.5, and 1 mg/mL). The SBMP inhibited the growth of both the Gram-positive bacterium, *R. erythropolis*, and the Gram-negative bacterium, *E. coli*, in liquid culture (Figure 8A,B). Both *R. erythropolis* and *E. coli* had roughly the same amount of GI (∼80%) at an SBMP concentration of 1 mg/mL. While the SBMP did inhibit some growth of the yeast *S. cerevisiae* (Figure 8C), the SBMP only inhibited 38% growth at a concentration of 1 mg/mL, indicating the SBMP has stronger antibacterial properties than antifungal properties. The antimicrobial activity of the SBMP could be attributed to the chemical moieties present in tannins and lignin, including phenolic and polyphenolic compounds, terpenoids, alkaloids, and hydroxide groups.^74,75^ Bioactive plant extracts are known to contain a mixture of these groups, and they can inhibit several bacterial mechanisms resulting in protein inactivation, decreased membrane integrity, efflux pump inhibition, and disruption of biofilm formation.^75^ Lignins are also known to have antifungal properties. Organosolv-extracted lignins from spruce displayed moderate GI (50 – 75%) against *Aspergillus niger* compared to kraft-extracted lignins from spruce, which displayed optimal GI (75–100%).^70^ The difference between GI percentages was attributed to the higher percentage of carbohydrates in the organosolv spruce lignins (9.7%) compared to the kraft spruce lignins (2.7%).^68^ The presence of polysaccharides in SBMP could also decrease its antifungal activity. The presence of the antibacterial and antifungal activity from the SBMP suggests it could hinder microbial growth on medical implants or other devices.

### Cell Viability

**Figure 9:**
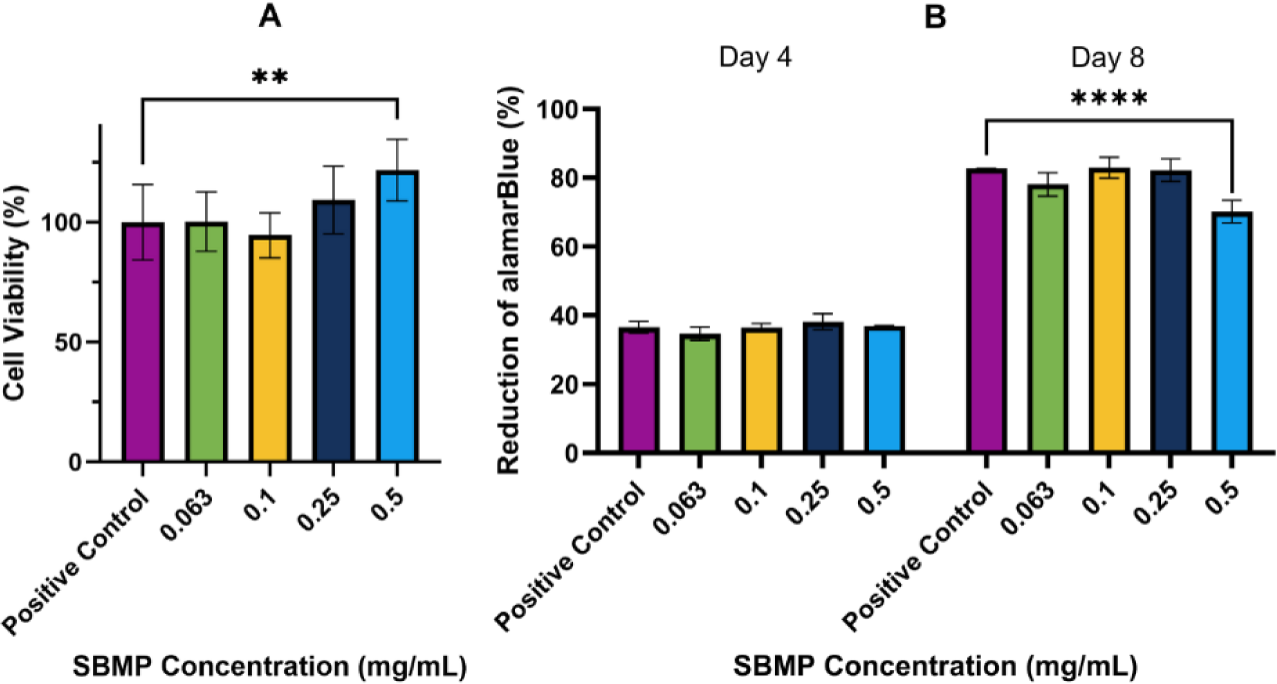
Cell viability in different concentrations of the SBMP (0.063, 01, 0.25, and 0.5 mg/mL): the percent cell viability determined with the MTT assay after 48 h (A) and percent reduction of alamarBlue after 4 and 8 days (B). Values represent mean ± standard deviation (*n* = 4). **p ≤ 0.01 and ****p ≤ 0.0001.

For SBMP to be suitable for biomedical applications, it cannot cause cell lysis or induce cell death in human cells. Two different assays were used to assess the cytotoxicity and cell viability of SBMP: the MTT assay and the alamarBlue assay. In the MTT assay, a water-soluble yellow tetrazolium salt is easily taken up by viable cells. Metabolically active cells reduce the tetrazolium salt into formazan (a purple color) using mitochondrial succinate dehydrogenases.^76^ The SBMP’s impact on cellular viability was assessed by measuring formazan production in the presence of the SBMP and comparing it to formazan production by untreated cells. If cell viability decreases to less than 70% in the presence of a compound, it is deemed cytotoxic.^77^ SBMP concentrations up to 0.5 mg/mL were not cytotoxic to the tested human adipose-derived stem cells (ADSC), and the concentrations up to 0.25 mg/mL showed no significant difference from the control (Figure 9A).

While the SBMP did not induce cell death, it could inhibit cell growth. To evaluate the impact of the SBMP on cell growth over time, the alamarBlue assay was used. In this assay, viable cells produce dehydrogenase enzymes which reduce resazurin (blue) to resorufin (pink), with higher reduction rates correlating with greater cellular activity and higher cellular viability.^78^ Resazurin levels were measured after 4 and 8 days of culture. After 4 days, samples containing 0.063, 0.1, 0.25, and 0.5 mg/mL of the SBMP showed similar alamarBlue (resazurin) reduction as the control, indicating that the SBMP did not inhibit cell growth (Figure 9B). After 8 days, a statistically significant difference in alamarBlue reduction was observed between the positive control (82.7% reduction) and the 0.5 mg/mL SBMP samples (70.1% reduction) (Figure 9B). These results indicate that SBMP concentrations of 0.25 mg/mL or lower had no effect on cell growth and proliferation of ADSC for up to 8 days, while the 0.5 mg/mL sample affected only cell growth and proliferation after 4 days of culture. The results for both assays suggest that SBMP concentrations below 0.5 mg/mL are not cytotoxic and will not hinder cellular growth, indicating that it has potential uses in biomedical applications.

## Conclusions

Utilizing agricultural byproducts, such as SBM, for medical, cosmetic, and coating applications provides a sustainable approach to reducing agricultural waste while leveraging the natural bioactive properties of this byproduct. To understand the potential applications of the SBMP, the SBMP was first isolated from SBM through a seven-day dialysis process. Then, the SBMP was chemically characterized using MALDI-TOF, FTIR, XPS, ^1^H NMR, and ^13^C NMR. These results identified a variety of compounds within the SBMP akin to lignins, tannins, and polysaccharides. The detected functional groups suggested that SBMP possesses biological activity, as demonstrated by the antioxidant assay. The phenolic and hydroxide groups confirmed by FTIR, XPS, ^1^H NMR, and ^13^C NMR explain the relatively high RSA (∼80%) exhibited by the SBMP. The presence of these compounds also likely enhanced the SBMP’s antimicrobial activity. The SBMP exemplified high antimicrobial activity against both Gram-positive and Gram-negative bacteria, while showing moderate inhibition of fungal growth. The inherent antioxidant and antimicrobial activities make SBMP a promising candidate for different biomaterial applications. This potential is further supported by the data showing that SBMP concentrations up to 0.5 mg/mL are noncytotoxic to ADSCs and support healthy cellular growth. Future research will focus on further characterizing the specific molecules isolated from SBM. Additional studies will also investigate whether SBMP maintains its biological activities in different biomaterial compositions.
